# Statistical Design of Biocarbon Reinforced Sustainable Composites from Blends of Polyphthalamide (PPA) and Polyamide 4,10 (PA410)

**DOI:** 10.3390/molecules26175387

**Published:** 2021-09-04

**Authors:** Mateo Gonzalez de Gortari, Manjusri Misra, Stefano Gregori, Amar K. Mohanty

**Affiliations:** 1 School of Engineering, Thornbrough Building, University of Guelph, Guelph, ON N1G 2W1, Canada; mgonza07@uoguelph.ca (M.G.d.G.); sgregori@uoguelph.ca (S.G.); mohanty@uoguelph.ca (A.K.M.); 2 Bioproducts Discovery and Development Centre, Department of Plant Agriculture, Crop Science Building, University of Guelph, Guelph, ON N1G 2W1, Canada

**Keywords:** polyphthalamide, polyamide 4,10, biocarbon, optimization, design of experiments

## Abstract

A full factorial design with four factors (the ratio of polyphthalamide (PPA) and polyamide 4,10 (PA410) in the polymer matrix, content percent of biocarbon (BioC), the temperature at which it was pyrolyzed and the presence of a chain extender (CE)), each factor with two levels (high and low), was carried out to optimize the mechanical properties of the resulting composites. After applying a linear model, changes in tensile strength, elongation at break and impact energy were not statistically significant within the considered material space, while the ones in the flexural modulus, the tensile modulus, density and heat deflection temperature (HDT) were. The two most influential factors were the content of BioC and its pyrolysis temperature, followed by the content of PPA. The affinity of PPA with a high-temperature biocarbon and the affinity of PA410 with a lower-temperature biocarbon, appear to explain the mechanical properties of the resulting composites. The study also revealed that the addition of CE hindered the mechanical properties. By maximizing the flexural modulus, tensile modulus and HDT, while minimizing the density, the optimal composite predicted is an 80 [PPA:PA410 (25:75)] wt% polymer composite, with 20 wt% of a BioC, pyrolyzed at a calculated 823 °C.

## 1. Introduction

To reduce the impacts that global climate change will have on human life, global warming must not exceed 1.5 °C. This means that many industries will have to adapt and change in order to reduce their carbon dioxide emissions [1]. The automotive industry is no exception and several strategies that have been recommended are currently being implemented, or developed [2]. Among these is fuel efficiency as it can be used as a short-term strategy that would reduce fuel demand at a low cost [2].

This trend started in the 1960s, and has been partially accomplished by replacing metal parts in vehicles with plastic ones [2]. The U.S. Environmental Protection Agency (EPA) has reported that practical CO_2_ emissions have decreased from 500 g/mile [311 g/km] of gasoline in the 1980s, to less than 400 g/mile [249 g/km] in the 2010s [3]. The top 14 car manufacturers evaluated by the EPA (which represent 98% of all produced vehicles) showed an increase in fuel economy (miles per gallon) in the period evaluated from 2014 to 2019 [3]. Plastic parts also have the advantage that they require less energy to be produced. For example, Ribeiro et al., show how in a multi-part braking system, the alternative of using glass fiber-reinforced plastics versus the current steel part results in a total reduction of use in energy, both during its production and use [4].

One of the plastics that has been successful in substituting metal parts is called polyphthalamide (PPA). Unlike many other polymers, PPA is a series or family of polymers, rather than a singular structure, derived from the condensation reaction of a mix of terephthalic and isophthalic acid, with a series of linear and branched diamines [5]. It has melting temperatures ranging from 300 to 325 °C, a heat deflection temperature (HDT) of around 125 °C and a tensile modulus between 2.5 and 3.5 GPa, depending on the diamines and ratio of terephthalic/isophthalic acid employed in its configuration [5]. It can also withstand a number of chemical attacks at normal temperatures from common solvents, such as glycols and alcohols [5]. However, it has the disadvantage that most of its production is based on non-renewable sources. A strategy to increase the sustainability of this type of polymer is to blend them with more renewable ones, as well as employing sustainable fillers that will not affect the properties of the resulting composites. Polyamide 4,10 (PA410) is a recently developed polymer (in the timescale of plastics) derived from castor oil (decanedioic acid) and butanediamine, which is normally sourced from petroleum, but can also be derived from succinic acid, as reported by the U.S. Department of Energy [6]. According to DSM, its manufacturer, 70% of the carbon in this polymer can be sourced from biological sources [7].

We developed the strategy of blending PPA with PA410 to reduce the amount of PPA required, lowering energy requirements and even improving thermomechanical properties. However, though PA 410 is a bio-derived material, it is also expensive. Adding adequate fillers that do not significantly increase the density or reduce the mechanical properties of the resulting polymers has been a challenge as well. Of the many fillers currently being studied, biocarbon has become more and more relevant and used. Derived from the solid char residue obtained from a biomass that went through pyrolysis [8,9], biocarbon has been studied both in isolation and as part of composites in a significant number of studies in our laboratory (as well as in other research groups) [10,11,12,13,14,15,16,17,18]. Other applications, such as the adsorption of water contaminants, also exist [19]. Biocarbon has many characteristics that affect its behaviour, including its particle size, biomass from which it was derived, as well as temperature at which it was pyrolyzed [8]. Thus, obtaining a biocarbon with optimized characteristics can be a challenge, depending on the application and polymer matrix being employed.

The first instinct of research investigating a new material space might be to conduct what is called a trial-and-error strategy. That is, the researcher starts by combining all the desired materials and modifying the content or properties of an individual component, until an “optimum” is found, to then modify the content or characteristics of another component. However, this risks the possibility of only finding a local optimum. Currently, there are a myriad of techniques to avoid this issue, and optimization studies can be found in many modalities and fields. High-throughput techniques promise to give advantages to companies and industries, over those who do not perform any optimization techniques [20], life cycle analysis metrics can be incorporated with optimization techniques, thus obtaining materials that can balance reducing environmental impact and increasing mechanical properties [21], and optimization techniques aided by machine learning are likely to change materials science significantly [22]. Thus, the fundamental importance of optimization studies becomes clear. 

**Figure 1:**
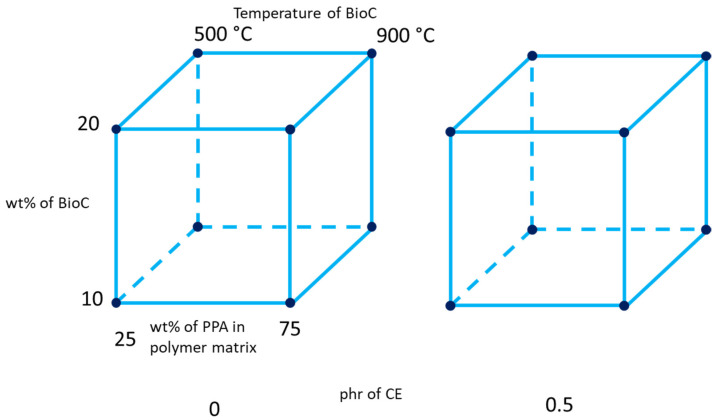
Design of experiment scheme, depicting the four factors: The ratio of PPA in the polymer matrix, wt% content of BioC, the temperature at which the BioC was pyrolyzed, as well as the presence or absence of 0.5 parts per hundred (phr) of CE.

To take advantage of fundamental optimization techniques and avoid reaching only local optimums within the considered material space, a full factorial design was chosen for this study. Four factors with two levels were chosen: high and low content of PPA in the polymer matrix with respect to the complementary PA410, high and low content of biocarbon, the presence or absence of a chain extender [CE] and a high and low temperature at which the biocarbon was produced. The full factorial analysis was chosen to learn of any interaction that could affect any of the measured responses, as fractional designs can confound important interactions. A scheme of the 2^4^ full factorial design can be seen in Figure 1.

## 2. Results and Discussion

Table 1 shows the results of all the mechanical properties of the composites. As it can be seen, properties like tensile strength and impact energy do not appear to have changed significantly between the different composites. The statistical analysis bears this, as seen in Table 2, as the fit for each model measured by R^2^ is less than 60% for tensile strength, elongation at break and the flexural modulus. This means that they were not considered relevant for further analysis, as the mean of all the values was a better predictor than the model itself.

**Figure 2:**
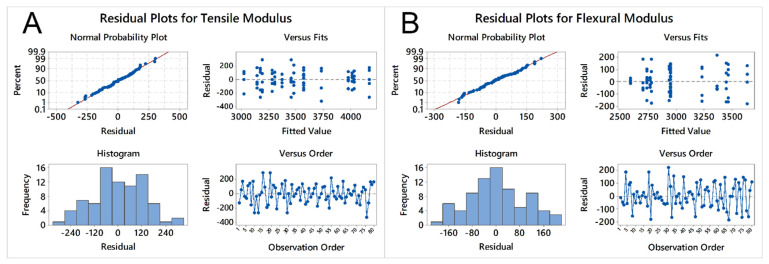
Residual plots for (**A**) tensile and (**B**) flexural modulus.

Before any analysis of the model results can be performed, the residuals of the different fitted values must be evaluated, in order to know if they follow a normal distribution. If no patterns in the residuals can be discerned, the linear model can be considered valid. In the case of the tensile and flexural modulus, as seen in Figure 2, the distribution of the residuals versus the fitted values behaves as a normal distribution, e.g., no particular pattern can be seen or discerned in any of the plots, in terms of fitted values, frequency or observation order.

**Figure 3:**
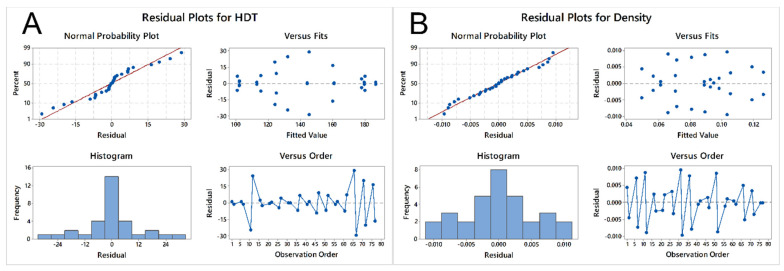
Residual plots for (**A**) HDT and (**B**) density.

However, in the case of HDT and density, as seen in Figure 3, the normal probability plots show distributions with distinct tails at both ends. In the case of HDT, a characteristic “S” pattern can be seen, while in the case of density, the outliers at both tail ends are more numerous in the normal probability plot, compared to the same plot for the flexural and tensile modulus. The fitted values also appear to show a pattern for HDT, as there are less differences in the extremes than in the tail ends.

**Figure A1:**
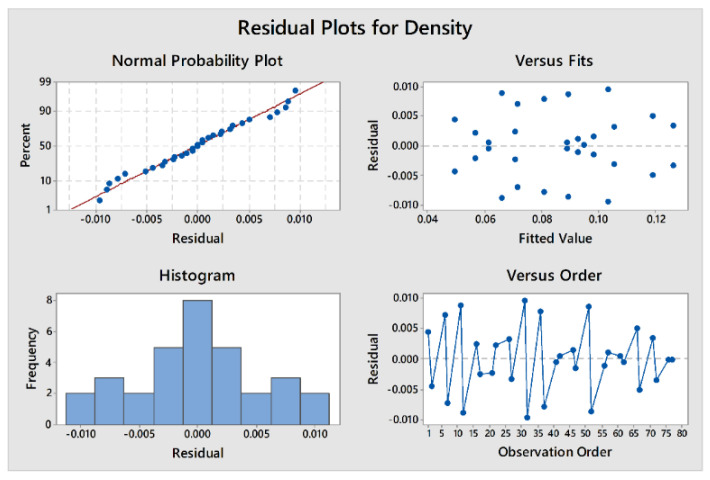
Residual plots for density, after a Box–Cox transformation.

There are many techniques that can convert data from a non-linear model to a linear one. That is, these techniques will improve the fit of the model and ensure normal distribution of the residuals, with respect to the newly transformed and fitted model. In the paper by Box and Cox [23], a method was presented whereby a parameter called lambda (*λ*) is chosen, selecting the most appropriate transformation for the data, based on a maximum value of lambda. This is shown in Equation (1):

(1) $$y_{i}^{\left(\lambda \right)}=\{\begin{array}{c} \frac{y_{i}^{\lambda }-1}{\lambda }\;\mathrm{if}\;\lambda \neq 0\\ \ln\left(y_{i}\right)\;\mathrm{if}\;\lambda =0 \end{array}$$

where $y_{i}^{\left(\lambda \right)}$ is the *i*^th^ response variable and how it should be transformed, according to the specific value of *λ*. Although this method can be applied by hand, modern computers can calculate and estimate the maximum value of λ numerically, in order to reach an optimum transformation. In the case of Minitab (the software used to analyze the results of the composites), it is an option that can be selected when applying a DOE analysis on the data. The results of these transformations are shown in Table 3. In the case of HDT, despite its “S” shape, not transforming the data is better than choosing a particular transformation. In the case of density, the transformation suggests that the model should use density to its fifth power. However, in statistics, it is often more adequate to choose the linear regression model, even if the residuals do not completely fit a normal distribution, especially if there is no compelling scientific reason, underlying scientific law or hypothesis that would explain why the density should have this behaviour with the studied factors. Additionally, the improvement seen in the fit for density is not significant enough that it would justify choosing the transformed model, rather than the linear model. Finally, looking at Figure A1, found in Appendix A, the residuals themselves are still not behaving according to a normal distribution, even after the proposed Box–Cox transformation. Thus, for HDT and density, we will use and analyze the linear model to understand the effects that the factors considered in this material space have on the response variables.

### 2.1. Analysis of Models

**Figure 4:**
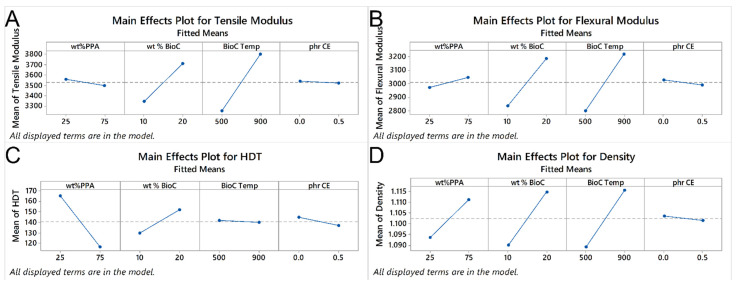
Main effects that the four different factors, wt% PPA, wt% BioC, the temperature of pyrolysis of BioC and the presence of chain extender, have on (**A**) tensile modulus, (**B**) flexural modulus, (**C**) HDT and (**D**) density.

**Figure 5:**
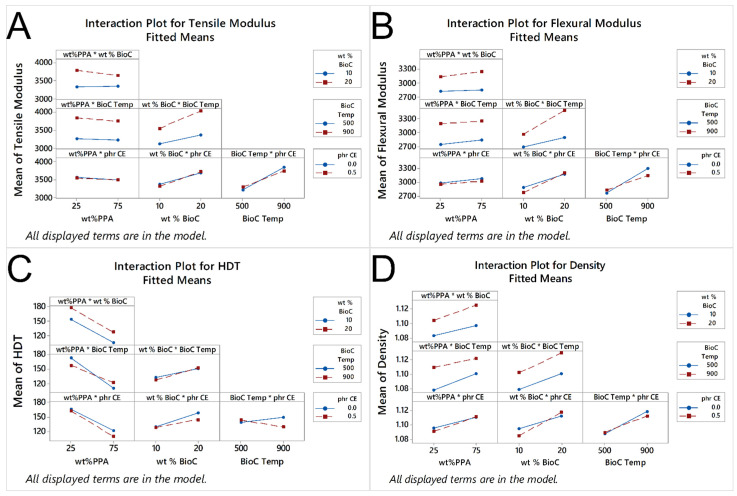
Interaction plots for the effects that the interactions between the main factors of wt% PPA, wt% BioC, and the temperature of pyrolysis of BioC have on (**A**) tensile modulus, (**B**) flexural modulus, (**C**) HDT and (**D**) density.

From the results in Table 2, we can see that the main factors that are statistically significant for the tensile modulus are the wt% of BioC and the temperature of pyrolysis of BioC. For two-way interactions, the significant ones are the ones between the wt% of PPA and BioC; the wt% of BioC and temperature of the pyrolysis of BioC; and the temperature of the pyrolysis of BioC and the presence of the chain extender. Finally, the three-way interaction between wt% of PPA, the temperature of the pyrolysis of BioC and the presence of the chain extender is also statistically significant. The main effects plot bears this, as seen in Figure 4A, there is a significant increase in the tensile modulus, when both the content of BioC and its pyrolysis temperature increase. The interaction plots in Figure 5A show a weaker, though still statistically significant effect, as the increase on the modulus is higher without CE. The tensile modulus is a measurement of how stiff a material is, so these results show that this property, within this material space, was not as dependent on the polymer matrix itself, but rather on the content of BioC. Clearly, a higher percentage of a BioC pyrolyzed at a higher temperature gives the best result in terms of increasing the tensile modulus. A possible explanation is that due to the presence of the BioC, (which increases the properties of the PPA in composites [18]) and BioC itself having a high tensile modulus compared to polymers, it acts as a stress concentrator and lends the resulting composites a higher stiffness. Biocarbon has also been shown to have a mix of graphitic and turbostratic functionality [24,25,26,27], having fewer functional groups, as the temperature at which it is pyrolyzed increases [28]. Biocarbon also increases the tensile modulus in other polymer matrices [12,29]. If the content of BioC was further increased, this relationship might change, as there might not be enough PPA to interact with the BioC. It is also possible that with a higher filler content, the BioC would also start agglomerating. Thus, if the materials were tested, the BioC would stop acting as a stress concentrator, and what could happen is that the different structures/sheets of BioC could start slipping from each other, thus decreasing the tensile modulus of the composite.

For the flexural modulus, as seen in Table 2, the main factors of content of PPA, BioC and the pyrolysis temperature of BioC, are all statistically significant. In the case of two-way interactions, the ones that are statistically significant are the ones between the BioC content and its pyrolysis temperature; the content of BioC and the presence of CE; and the temperature of pyrolysis of biocarbon and the presence of CE. The three-way interaction of the content of PPA, the temperature of BioC and the amount of BioC is also significant, as well as the three-way interaction between the content of PPA, the content of BioC and the presence of the CE; as well as the three-way interaction between the content of PPA, the temperature of pyrolysis of BioC and the presence of CE. Finally, the four-way interaction between the content of PPA and BioC, the temperature of pyrolysis of BioC and the presence of CE is also statistically significant. Figure 4B shows that a higher content of PPA and BioC, with BioC pyrolyzed at a higher temperature, increases the flexural modulus, while the presence of CE has a slight negative effect. In terms of interactions, we can see that a higher content of BioC, with BioC pyrolyzed at a higher temperature, resulted in a higher flexural modulus, while the presence of CE interacted negatively with both the content of PPA and BioC. The same stiffening effect, previously seen in a PPA matrix [18], also applies to the flexural modulus.

The question arises as to why the CE did not improve these two mechanical properties, compared to preliminary testing and blends. Russo and Acierno found that the addition of a cross-linking agent actually worsened the creep properties of a PPA-based, 50 wt% glass fiber-filled composite [30], which they attributed to non-optimized processing parameters. In the case of this study, the processing parameters were kept the same for all composites, to reduce the number of factors, as well as sources of error. Therefore, it is possible that each individual composite was not processed under ideal conditions, and that the CE agglomerated. Other possibilities include poor bonding with the biocarbon, or with the polymer matrix, or both.

In the case of HDT, Table 2 shows that the only statistically significant factors were the content of PPA, the content of BioC and the two-way interaction of the content of PPA with the temperature of pyrolysis of BioC. As the content of PPA increased, the HDT decreased, while as the content of BioC increased, so did the HDT, as seen in Figure 4C. In the interaction of PPA content with the temperature, as seen in Figure 5C, the higher content of PPA with the BioC pyrolyzed at a higher temperature resulted in a higher HDT, with the opposite happening with a low content of PPA. Watt et al. found that the addition of a high-temperature biocarbon (900 °C) had a higher increase in the HDT of a PA410 matrix than a low temperature one [31]. They attributed this to the fact that a higher temperature biocarbon has a higher carbonaceous content, which means that the structure is stiffer. They also found that PA410 had a better interaction with a low temperature biocarbon (300 °C), possibly due to hydrogen bonding between the biocarbon and the polymer matrix. Our statistical model bears this, as a higher content of PA410 with the low temperature BioC, results in a higher HDT. As the neat PA410 has a higher HDT than PPA, it stands to reason that to improve this characteristic, the temperature of pyrolysis of BioC should be low.

Finally, for density, the statistically significant factors are the content of PPA, the content of BioC and the temperature of pyrolysis of BioC. The two-way interaction that is statistically significant is the content of BioC and the presence of CE; as is the three-way interaction between the content of PPA, the content of BioC and the presence of CE; as well as the three-way interaction between the content of PPA, the temperature of pyrolysis of BioC and the presence of the CE, as seen in Table 2. From Figure 4D, we can see that as both the content of PPA and BioC increase, so does the density. Figure 5D shows that with the BioC pyrolyzed at a high temperature, there is a significant increase in density as the content of PPA increases, compared to a lower increase with low-temperature BioC. Figure 5D also shows that the presence of the CE decreases the density of a composite with a higher content of BioC, while the absence of the CE makes it lower. As previously stated, the main phenomena that appears to govern the mechanical properties of the produced composites is the affinity and interaction between the BioC pyrolyzed at a high temperature and the PPA matrix, as well as the non-affinity of the CE within this material space. The density of neat PPA is higher than that of PA410, so it stands to reason that all things being equal, a higher content of PPA would yield a composite with higher density. If the BioC pyrolyzed at a higher temperature has a better affinity with PPA, then it is possible that a larger amount of biocarbon could be packed within the composite, explaining why the density increases with the higher content of BioC and PPA.

### 2.2. Optimization

**Figure 6:**
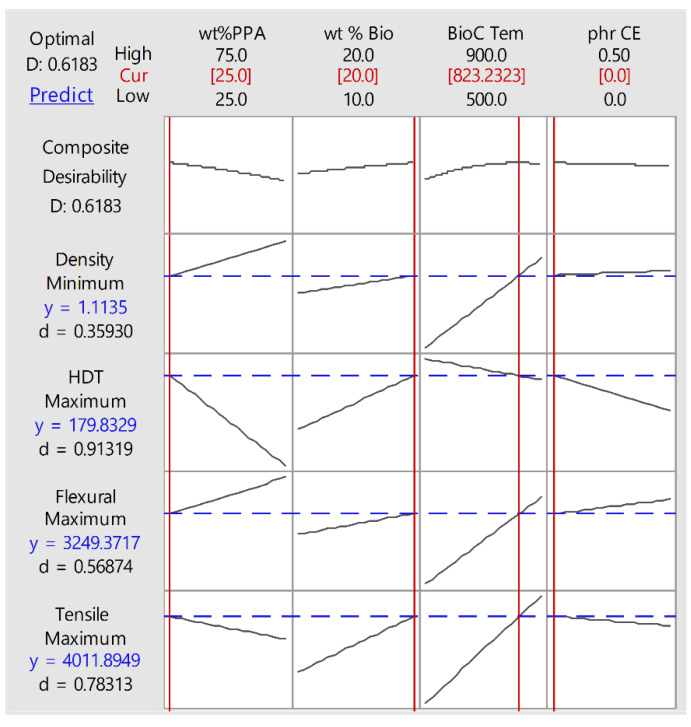
Optimization plot of material space considered.

One of the main interests of this paper is optimizing the mechanical properties of the composites, after establishing statistical methods that would allow us to predict the behaviour. The statistical analysis software can construct a model that allows the user to ask for the optimum composite under certain conditions. We limited the material space within the level of the factors already tested, so that there would be no extrapolation of data. We also set the program to maximize the flexural and tensile modulus, as well as the HDT, and to minimize the density. Additionally, the program asks for “Weight” and “Importance”. To avoid bias or skewing the prediction, we left all the values set at “1” for all factors. If a desired application or process required a specific value in a particular characteristic, the optimized composite would be different. The result of the optimization is shown in Figure 6.

The result shows that the optimum composite within the material space and the conditions imparted upon the program is an 80 (PPA:PA410 (25:75)) wt% composite, with 20 wt% of a BioC pyrolyzed at 823 °C, and no CE, though this composite would have to be produced and tested in order to validate the model. As seen during the analysis of the statistical models, in many cases, the amount of PPA had a negative or slightly positive effect on the properties, so it is logical that a composite with a low amount of this polymer would be selected. Higher content of BioC resulted in high mechanical properties, so despite the increase in density, it is also logical that 20 wt% would be chosen by the algorithm. The temperature of pyrolysis of BioC also makes sense, as we saw that even at a low percentage of PPA, the BioC content enhances all mechanical properties. Finally, the presence of CE hindered the mechanical properties in all categories, so it is logical that it is absent from this optimization.

## 3. Materials and Methods

PPA, grade Zytel (grade: HTFNFE8200 NC010, Dupont, Wilmington, DE, USA) and PA 4,10 (grade: Ecopaxx Q210E-H, DSM, Heerlen, Netherlands) were dried for 4 h and 24 h, respectively, at 80 °C, in order to ensure the moisture content did not exceed the level recommended by the manufacturers (0.1 and 0.15%, respectively). The biocarbon was produced from pine wood saw dust (Pine: 4026, American Wood Fibers, Columbia, MD, USA), by drying the material for at least 48 h at 80 °C, then pyrolyzed using a GLO 0/11-1G batch type industrial front-loading retort furnace (Carbolite Gero Limited, Hope Valley, United Kingdom) at 500 and 900 °C, respectively, using a heating rate of 5 °C/min, and a dwelling time of 1 h for both temperatures. After the removal of the pyrolyzed material from the furnace, the material was reduced in size using a commercial blender, and was then ball-milled for 2 h, using zirconium oxide vessels, each one utilizing 100 ceramic zirconium oxide balls and placing 52 g of the biocarbon in each one. The biocarbon was then ball-milled at 200 rpm, reversing the direction of the milling every half hour, for a total of 2 h. This material is what is denominated as BioC. Afterwards, BioC was dried at 80 °C for the 900 °C BioC or at 60 °C for the 500 °C BioC, for at least 24 h, before the processed blends were extruded.

All the blends were weighted and pre-measured, to be hand mixed, according to the composition stipulated in Table 4. After hand mixing the material together, they were fed into a twin-screw extruder machine (Micro-27/GL-48D, Leistritz Advanced Technologies Corp, Nurnberg, Germany) at a speed of 5 kg/hr via a screw feeder. The machine had 12 heating zones, whereby the first zone of the machine was set to a temperature of 165 °C, zones 2 and 3 to 300 °C, zones 4 to 11 to 305 °C, and 300 °C at the final die zone. The resulting strands, after being cooled in a water bath, were pelletized. The pellets, after being dried at 75 °C for at least 20 h to remove residual moisture, were processed into testable samples with a Mini Jector model #55 (Miniature Plastic Molding, Farmington Hills, MI, USA), with a rear barrel temperature of 282 °C, a front barrel temperature of 288 °C and a nozzle temperature of 302 °C. The molten polymer was injected into molds heated up to and kept at 100 °C, based on the recommended molding conditions of PPA. The order in which the composites were processed was randomized to reduce any bias or noise in the results. The run order can be seen in Table 4.

### 3.1. Mechanical Properties

The resulting samples, after being conditioned for at least 40 h, were tested for their flexural and tensile properties utilizing a universal testing machine 3382 (Instron, Norwood, MA, USA), under the ASTM protocols D638-14 and D790-15, respectively. The tensile crosshead speed was 5 mm/min for all composites, while the flexural speed was 14 mm/min. The impact energy tests were run under ASTM D256, using notched samples, with an Izod pendulum, a model Zwick/Roell Impact tester (Ulm, Germany). In all these studies, a total of 5 samples were utilized. Density was measured using an electronic Densimeter MDS-3000 (AlfaMirage, Osaka, Japan), taking the average of two samples.

### 3.2. HDT (Heat Deflection Temperature)

For HDT, the machine DMA Q500 (TA Instruments, New Castle, DE, USA) was used, with each sample being tested with a force calculated to exert 0.455 MPa of stress unto the sample, using the average of two samples, heating the samples at a rate of 2 °C/min, until a 0.22% strain was measured by the machine. The HDT measurement was taken at 0.2% strain.

### 3.3. Design of Experiments Methodology

As these composites are novel, all processing conditions and parameters were kept the same, to measure the influence that each of the two-level factors had on the mechanical and thermal properties of the composites, without any interference from the processing conditions. This also helps avoid doubling the required number of composites, to keep a full factorial design, if a single of the processing conditions was varied.

The program Minitab 17 was employed, utilizing ANOVA and interaction plots. ANOVA allows us to test multiple hypotheses for each property, in which the null hypothesis (i.e., the factor or combination of factors does not affect the property) is tested against the alternative hypothesis (i.e., that the factor or combination of factors does affect the property). A typical significance value of *p* ≤ 0.05 was used, that is, the alternative hypothesis cannot be rejected if this value is equal or less than 0.05. Each measurement of a composite was treated as a block, to increase the statistical power of the analysis. A linear model with all their factors and interactions was used initially for all the measured mechanical properties. Interaction plots were also calculated to see the effect of each factor on each property, as well as residual plots to check the normal distribution of the residuals versus the fitted values. The response variables analyzed were the tensile strength and modulus, the elongation at break, the flexural modulus, the impact energy, the HDT and the density of the composites.

A central composite design could have been used to further estimate the optimum conditions if a nonlinear relationship is detected during the full factorial analysis. However, the required axial points could point to regions that would be outside the material space, such as using 0% content of BioC with two different temperatures. Box–Behnken designs can also be used for nonlinear relationships, which again, were not detected in the data considered for this paper. They also require three levels per factor, which would significantly increase the number of runs, leading to a fractional factorial design, with the disadvantage of confusing higher-order interactions.

## 4. Conclusions

Of all the mechanical properties evaluated for the material space composed by wt% of PPA and BioC, the temperature at which BioC was pyrolyzed and the presence or absence of 0.5 phr of CE, only the tensile and flexural modulus as well as the HDT and density were affected in a statistically significant manner by the chosen factors. The linear model was considered adequate for all the response variables, despite the non-normal distribution of the residuals in the HDT and density models. Applying a Box–Cox transformation to HDT yielded an untransformed model. In the case of density, though it suggested transforming the linear model by substituting the response variable to the fifth power of the same variable, there was not enough of an increase in the explanation power for which to prefer it over the linear model.

The only main factor that was considered statistically significant in all the mechanical characteristics was the content of BioC, as each response variable had its own set of significant factors and significant interactions. The main interaction that was statistically significant in all the composite characteristics was the two-way interaction between the content of PPA and the content of BioC.

An optimized composite was predicted by giving equal weight and importance to all factors, with the composition of 80 (PPA:PA410 (25:75)) wt% composite, with 20 wt% of a BioC pyrolyzed at 823 °C, and no CE, with a predicted flexural modulus of 3.25 GPa, a tensile modulus of 4.01 GPa, a density of 1.11 g/cm^3^ and an HDT of 179 °C, at 0.455 MPa at a 0.2% strain.

The material space considered in this paper shows great potential for increasing the sustainability of pieces that require the use of a high temperature polymer like PPA, without compromising its mechanical properties, or increasing the density in a significant manner.

## Figures and Tables

**Table 1:** Results of tests carried out on all the produced composites.

wt% of Polymer Matrix (Ratio of %PPA/%PA410)	wt% of BioC (%)	BioC Production Temp. (°C)	Phr of CE	Tensile Strength (MPa)	Tensile Modulus (GPa)	% Elongation at Break	Flexural Modulus (GPa)	Impact Energy (J/m)	Density (g/cm^3^)	HDT at 0.455 MPa, 0.2% Strain (°C)
90 (25/75)	10	500	0.5	74.4 ± 1.84	3.137 ± 0.1184	6.74 ± 4.25	2.704 ± 0.106	49.36 ± 5.38.	1.064 ± 0.007	180.06 ± 1.48
90 (75/25)	10	500	0.5	76.3 ± 0.98	3.171 ± 0.2007	6.64 ± 2.12	2.734 ± 0.107	58.59 ± 13.07	1.089 ± 0.011	102.38 ± 2.03
90 (25/75)	10	900	0.5	73.5 ± 0.86	3.455 ± 0.2012	14.31 ± 3.72	2.736 ± 0.0335	45.12 ± 9.66	1.083 ± 0.013	132.27 ± 34.39
90 (75/25)	10	500	0	73.4 ± 1.13	3.146 ± 0.2302	5.24 ± 1.74	2.776 ± 0.1414	49.42 ± 6.49	1.088 ± 0.004	102.27 ± 3.03
90 (25/75)	10	500	0	72.0 ± 4.48	3.022 ± 0.1300	8.67 ± 5.10	2.588 ± 0.019	66.41 ± 18.08	1.073 ± 0.004	144.39 ± 0.86
80 (25/75)	20	900	0	76.7 ± 3.24	4.165 ± 0.1748	3.87 ± 0.93	3.365 ± 0.1231	49.04 ± 6.49	1.122 ± 0.004	178.14 ± 6.03
80 (25/75)	20	900	0.5	78.9 ± 1.48	4.032 ± 0.1001	5.60 ± 2.08	3.443 ± 0.1212	46.56 ± 10.13	1.120 ± 0.014	161.35 ± 0.35
80 (25/75)	20	500	0.5	81.3 ± 2.08	3.564 ± 0.1190	12.03 ± 6.16	2.944 ± 0.0929	65.02 ± 19.13	1.098 ± 0.011	180.19 ± 9.55
80 (25/75)	20	500	0	77.3 ± 1.04	3.367 ± 0.0809	4.79 ± 0.32	2.764 ± 0.03497	61.66 ± 7.60	1.078 ± 0.001	186.94 ± 1.44
80 (75/25)	20	500	0	79.8 ± 1.37	3.278 ± 0.1263	7.49 ± 0.94	2.935 ± 0.1204	60.49 ± 9.66	1.116 ± 0.002	125 ± 12.74
90 (75/25)	10	900	0.5	75.7 ± 2.21	3.477 ±0.1634	4.95 ± 1.25	2.948 ± 0.07224	60.02 ± 20.41	1.107 ± 0.0120	101.09 ± 9.54
80 (75/25)	20	500	0.5	78.2 ± 1.10	3.302 ± 0.0714	7.32 ± 1.77	2.949 ± 0.1059	63.91 ± 6.46	1.110 ± 0.001	113.03 ± 1.36
90 (75/25)	10	900	0	79.6 ± 1.19	3.566 ± 0.1192	5.89 ± 1.22	2.950 ± 0.1144	55.08 ± 7.61	1.107 ± 0.001	115.66 ± 9.96
80 (75/25)	20	900	0	79.8 ± 1.68	3.973 ± 0.0905	5.60 ± 1.77	3.633 ± 0.1162	67.08 ± 7.75	1.135 ± 0.006	145.89 ± 41.31
80 (75/25)	20	900	0.5	78.7 ± 1.20	4.013 ± 0.0975	5.65 ± 1.56	3.461 ± 0.1409	54.48 ± 4.39	1.141 ± 0.004	124.37 ± 28.07
90 (25/75)	10	900	0	78.4 ± 5.96	3.730 ± 0.2208	8.77 ± 6.79	3.229 ± 0.1290	46.32 ± 7.25	1.112 ± 0.000	160.19 ± 23.28

**Table 2:** Degrees of freedom, *p*-values, R^2^ fit of model for the mechanical properties.

Source	Tensile Strength		Tensile Modulus		Elongation at Break		Flexural Modulus		Impact Energy		HDT		Density	
	R^2^ = 59.36%		R^2^ = 87.65%		R^2^ = 45.97%		R^2^ = 91.40%		R^2^ = 36.28%		R^2^ = 85.57%		R^2^ = 94.34%	
	DF	*p*-Value	DF	*p*-Value	DF	*p*-Value	DF	*p*-Value	DF	*p*-Value	DF	*p*-Value	DF	*p*-Value
Model	15	0.000	15	0.000	15	0.000	15	0.000	15	0.007	15	0.000	15	0.000
Linear	4	0.000	4	0.000	4	0.007	4	0.000	4	0.003	4	0.000	4	0.000
wt% PPA	1	0.042	1	0.058	1	0.007	1	0.002	1	0.012	1	0.000	1	0.000
wt% BioC	1	0.000	1	0.000	1	0.131	1	0.000	1	0.015	1	0.002	1	0.000
BioC Temp	1	0.046	1	0.000	1	0.461	1	0.000	1	0.024	1	0.757	1	0.000
phr CE	1	0.993	1	0.599	1	0.029	1	0.093	1	0.823	1	0.208	1	0.418
2-Way Interactions	6	0.000	6	0.000	6	0.003	6	0.000	6	0.092	6	0.177	6	0.051
wt% PPA × wt% BioC	1	0.301	1	0.020	1	0.009	1	0.102	1	0.979	1	0.954	1	0.243
wt% PPA × BioC Temp	1	0.394	1	0.435	1	0.397	1	0.361	1	0.007	1	0.048	1	0.094
wt% PPA × phr CE	1	0.089	1	0.841	1	0.038	1	0.669	1	0.452	1	0.517	1	0.322
wt% BioC × BioC Temp	1	0.003	1	0.000	1	0.004	1	0.000	1	0.184	1	0.621	1	0.322
wt% BioC × phr CE	1	0.116	1	0.136	1	0.415	1	0.007	1	0.506	1	0.316	1	0.016
BioC Temp × phr CE	1	0.001	1	0.004	1	0.977	1	0.000	1	0.323	1	0.061	1	0.142
3-Way Interactions	4	0.006	4	0.047	4	0.062	4	0.000	4	0.166	4	0.625	4	0.029
wt% PPA × wt% BioC × BioC Temp	1	0.405	1	0.069	1	0.034	1	0.040	1	0.785	1	0.749	1	0.833
wt% PPA × wt% BioC × phr CE	1	0.017	1	0.834	1	0.309	1	0.000	1	0.070	1	0.804	1	0.016
wt% PPA × BioC Temp × phr CE	1	0.515	1	0.013	1	0.483	1	0.004	1	0.240	1	0.324	1	0.020
wt% BiocC × BioC Temp × phr CE	1	0.004	1	0.580	1	0.077	1	0.141	1	0.179	1	0.245	1	0.592
4-Way Interactions	1	0.640	1	0.917	1	0.009	1	0.000	1	0.930	1	0.334	1	0.501
wt% PPA × wt% BioC × BioC Temp × phr CE	1	0.640	1	0.917	1	0.009	1	0.000	1	0.930	1	0.334	1	0.501
Error	64		64		64		64		64		16		16	
Total	79		79		79		79		79		31		31	

**Table 3:** Parameters calculated for Box–Cox transformation of HDT and density.

Parameter Calculated for Box-Cox Transformation	HDT	Density
Rounded λ	1	5
Estimated λ	0.880092	4.9839
95% CI for λ	(−1.06641, 2.83559)	(−9.35660, 19.2824)
R^2^ before and after transformation	85.57% vs. 85.57%	94.34% vs. 94.41
New model	$y_{i}^{\left(\lambda \right)}=y_{i}$	$y_{i}^{\left(\lambda \right)}=y_{i}^{5}$

**Table 4:** Coding and running order of the processing of the composites.

Run Order	Standard Order	wt% of Polymer Matrix (Ratio of %PPA/%PA410)	wt% of BioC	BioC Production Temp (°C)	Phr of Chain Extender
1	9	90 (25/75)	10	500	0.5
2	10	90 (75/25)	10	500	0.5
3	13	90 (25/75)	10	900	0.5
4	2	90 (75/25)	10	500	0
5	1	90 (25/75)	10	500	0
6	7	80 (25/75)	20	900	0
7	15	80 (25/75)	20	900	0.5
8	11	80 (25/75)	20	500	0.5
9	3	80 (25/75)	20	500	0
10	4	80 (75/25)	20	500	0
11	14	90 (75/25)	10	900	0.5
12	12	80 (75/25)	20	500	0.5
13	6	90 (75/25)	10	900	0
14	8	80 (75/25)	20	900	0
15	16	80 (75/25)	20	900	0.5
16	5	90 (25/75)	10	900	0

## Data Availability

The data presented in this study are available on request from the corresponding author.

## Sample Availability

Samples of the compounds are available from the authors.
